# Supplementary material for: Structurally diverse viral inhibitors converge on a shared mechanism to stall the antigen transporter TAP
Source: Proc Natl Acad Sci U S A. 2025 Sep 16;122(38):e2516676122. doi: 10.1073/pnas.2516676122 (PMC12478189; doi:10.1073/pnas.2516676122)
Supplement: Supplementary file 1 — Appendix 01 (PDF) [file pnas.2516676122.sapp.pdf]

PNAS

Supporting Information for
Structurally diverse viral inhibitors converge on a shared mechanism to stall the antigen transporter TAP

Authors: James Lee[1,2,4], Victor Manon[1,2,3,4], and Jue Chen[1,2*]

*Corresponding author: Dr. Jue Chen (Contact author)

Email: juechen@rockefeller.edu

This PDF file includes:

Figures S1 to S7
Data Table S1

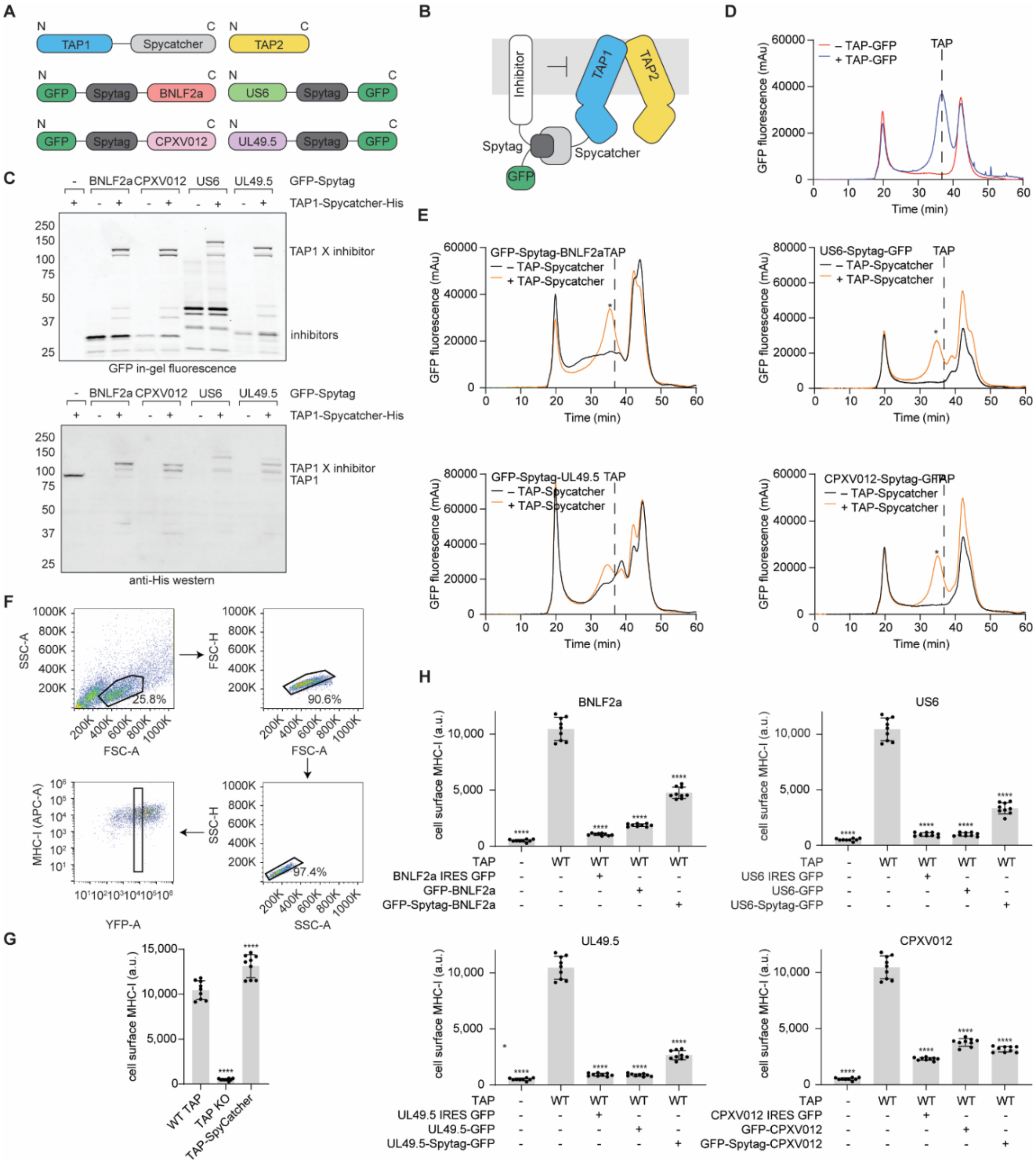

Supplemental Fig. 1. Capture of virally inhibited TAP complexes using the SpyCatcher-SpyTag system.

A. Schematic of the individual SpyCatcher- and SpyTag-labeled constructs. **B.** Schematic of the general SpyCatcher-SpyTag labeling strategy. **C.** The TAP/inhibitor complexes form efficiently in cells. SDS-PAGE of cell lysates expressing combinations of SpyCatcher-labeled TAP and different GFP-SpyTag-labeled viral inhibitors as visualized by in-gel fluorescence and Western Blot. **D.** TAP migrates as a monodisperse peak as judged by fluorescence size exclusion (FSEC). The peak corresponding to TAP is marked by a dashed line. **e.** The TAP/inhibitor complexes migrate as a monodisperse peak. The peak corresponding to TAP and the TAP/inhibitor complex are marked by a dashed line and asterisk, respectively. **F.** Representative FACS plots and gating strategy. Data shown are from HEK293S cells expressing YFP only. **G.** The TAP-SpyCatcher construct is

functional. Flow cytometry analysis of MHC-I cell surface levels in TAP knockout cells transduced with baculovirus expressing WT or SpyCatcher-tagged TAP. Data represent the mean fluorescence intensity and standard error of three technical replicates of three biological replicates (n=9). Error bars represent the SEM. Statistical significance relative to the TAP-Spycatcher sample was tested by one-way analysis of variance. ns, not significant; ****P<0.0001. **H.** The SpyTag-labeled viral inhibitors are functional. Flow cytometry analysis of MHC-I cell surface levels in TAP knockout cells transduced with baculovirus expressing combinations of TAP and the different viral inhibitors. Data represent the mean fluorescence intensity and SEM of three technical replicates of three biological replicates (n=9). Samples from TAP knockout (KO) cells and wild-type (WT) cells alone are replicated in each chart. Samples with SpyTag-GFP labelled viral inhibitors are replicated from Fig. 1b. Statistical significance relative to the wild-type was tested by one-way analysis of variance. ns, not significant; ****P<0.0001.

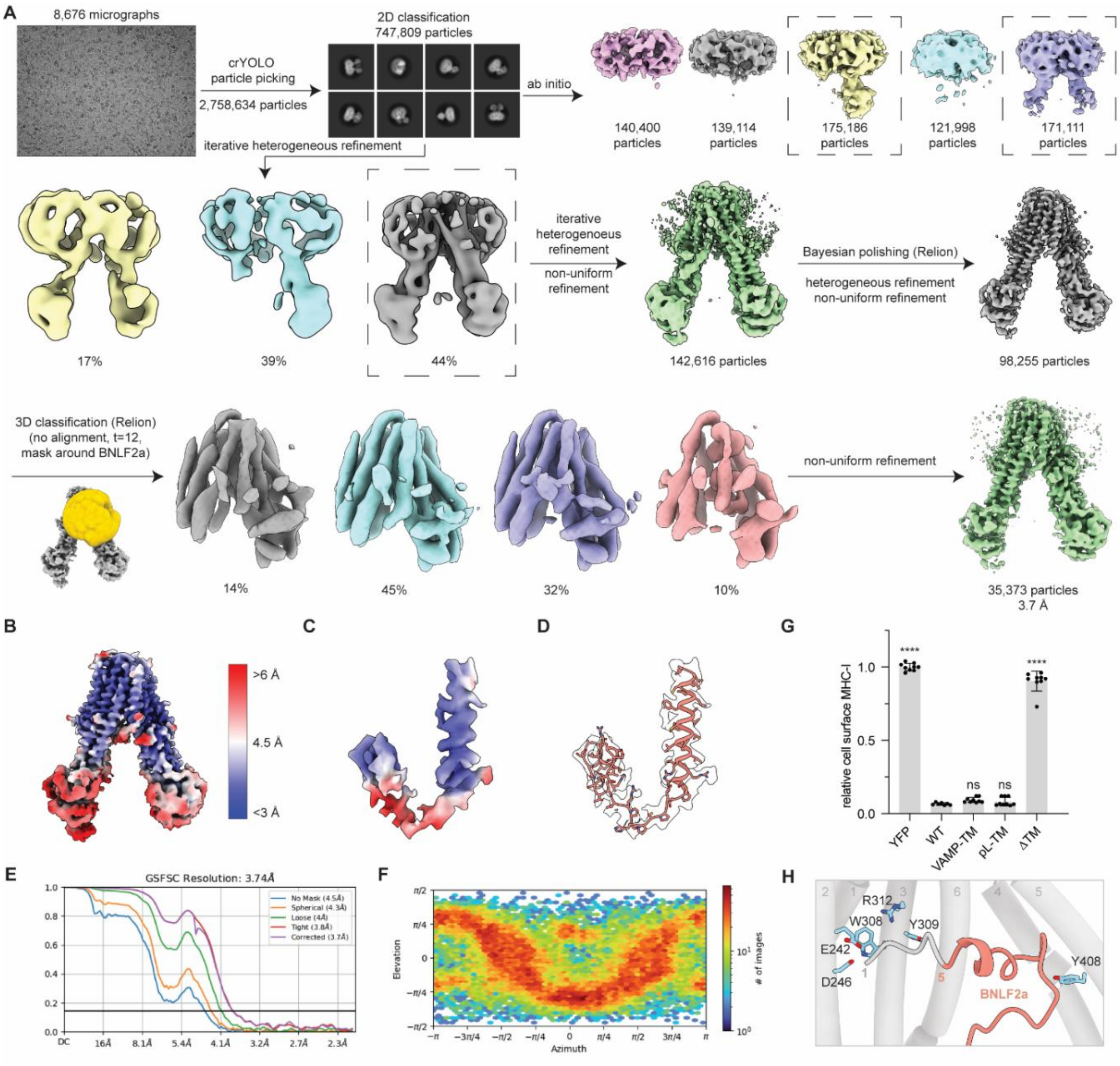

Supplemental Fig. 2. Structural and biochemical analysis of the TAP/BNLF2a complex.

A. Flowchart of the image processing of BNLF2a-inhibited TAP. **B.** Local resolution map of BNLF2a-inhibited TAP. **C.** Local resolution map of the BNLF2a. Reconstruction is contoured to 0.2 SDs. **D.** Map to model fit of BNLF2a. Reconstruction is contoured to 0.15 SDs. **E.** Fourier shell correlation (FSC) curves of the final reconstruction of BNLF2a-inhibited TAP. **F.** Angular distribution of the final reconstruction of BNLF2a-inhibited TAP. **G.** Flow cytometry analysis of the HEK293S cells expressing BNLF2a transmembrane helix swap variants coupled to YFP with an internal ribosomal entry site (IRES) element. Data represent the relative MHC-I cell surface levels normalized to the YFP only sample and represent three technical replicates of three biological replicates (n=9). Error bars represent the SEM. Statistical significance relative to the wild-type was tested by one-way analysis of variance. ns, not significant; ****P<0.0001. **H.** Putative binding mode of the BNLF2a N-terminus to the TAP N-pocket. Residues 1-5 of BNLF2a are not visible in the cryo-EM density and are putatively modeled.

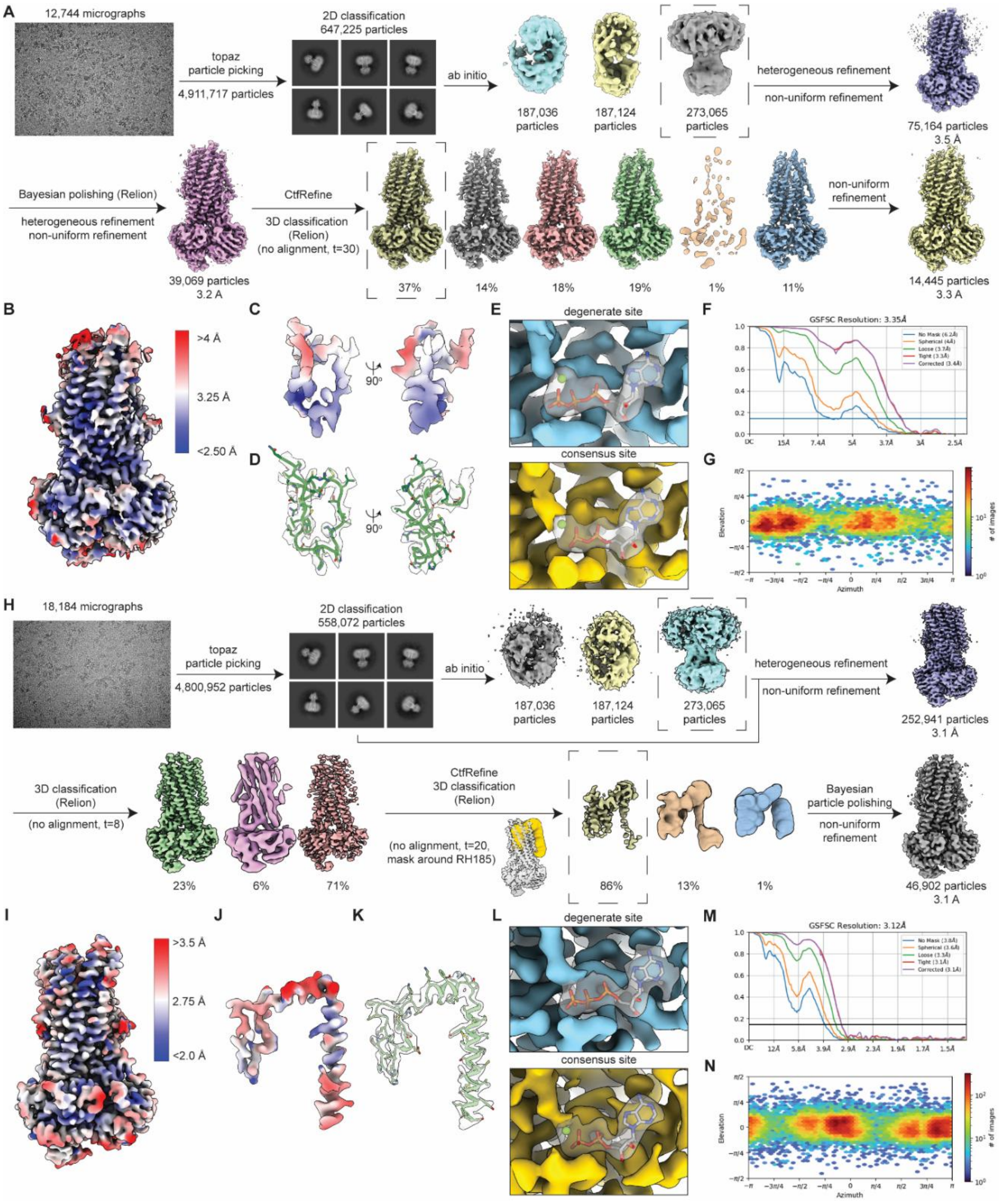

Supplemental Fig. 3. Cryo-EM processing of hUS6-inhibited and rhUS6-inhibited TAP structures.

A. Flowchart of the image processing of hUS6-inhibited TAP. **B.** Local resolution map of the final overall complex of hUS6-inhibited TAP. **C.** Local resolution map of hUS6. Reconstructions are contoured to 0.3 SDs. **D.** Map to model fit of hUS6. Reconstructions are contoured to 0.3 SDs. **E.** Map to model fit around the nucleotide binding sites of TAP. Reconstructions are contoured to 0.4 SDs. **F.** Fourier shell correlation (FSC) curves of the final reconstruction of hUS6-inhibited TAP. **G.** Angular distribution of the final reconstruction of hUS6-inhibited TAP. **H.** Flowchart of the image processing of rhUS6-inhibited TAP **I.** Local resolution map of the final overall complex of rhUS6-

inhibited TAP. **J.** Local resolution map. Reconstructions are contoured to 0.3 SDs. **K.** Map to model fit. **L.** Map to model fit around the nucleotide binding sites of TAP. Reconstructions are contoured to 0.3 SDs. **M.** Fourier shell correlation (FSC) curves of the final reconstruction of rhUS6-inhibited TAP. **N.** Angular distribution of the final reconstruction of rhUS6-inhibited TAP.

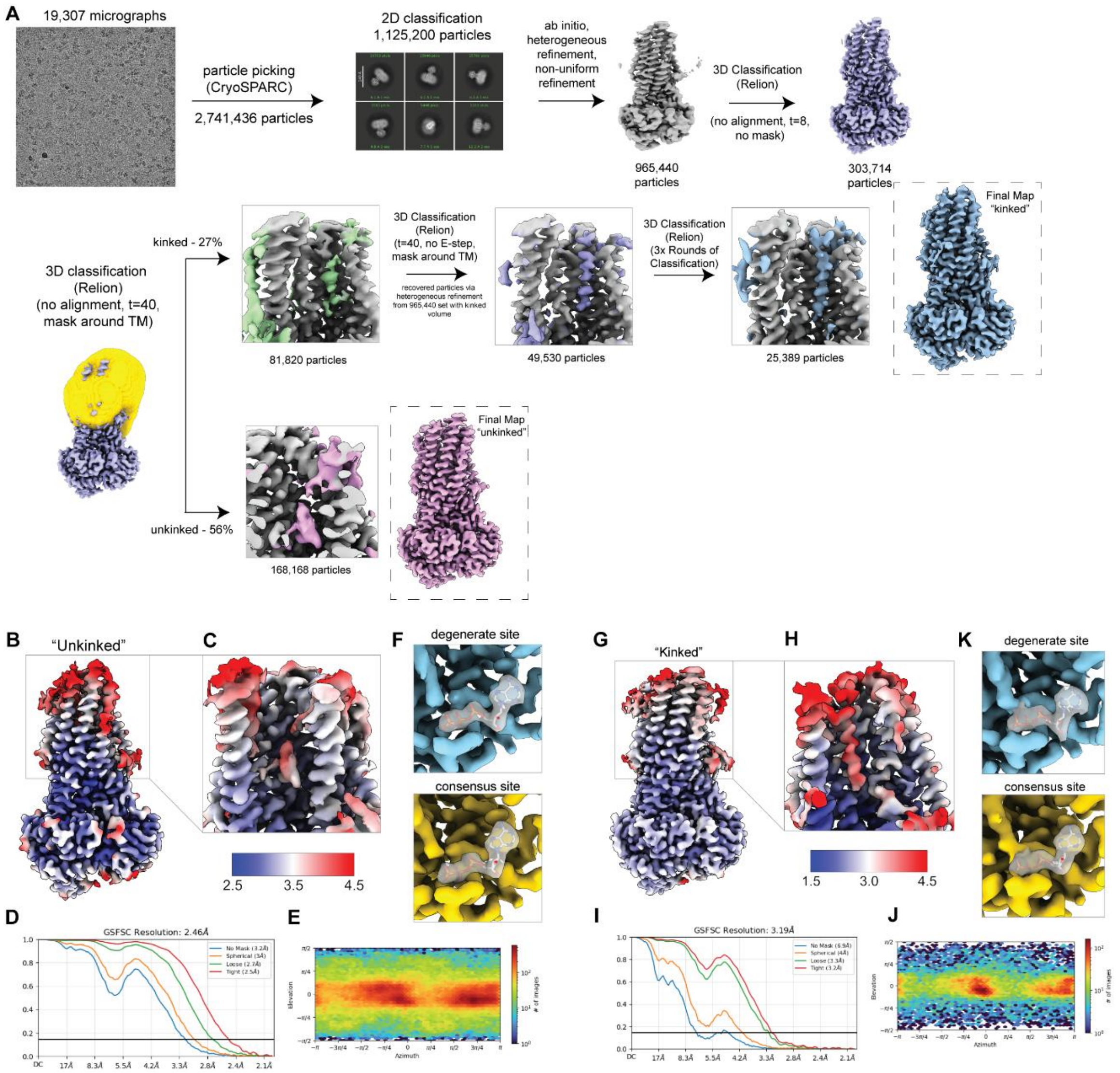

Supplemental Fig. 4. Cryo-EM processing of bUL49.5-inhibited TAP(EQ).

A. Flowchart of the image processing of bUL49.5-inhibited TAP. **B,C.** Local resolution map of bUL49.5-TAP(EQ) in the "unkinked" conformation. Reconstructions are contoured to 0.188 SDs. **D.** Fourier shell correlation (FSC) curves of the "unkinked". **E.** Angular distribution of the final reconstruction. **F.** Map to model fit around the nucleotide binding sites of TAP. Reconstructions are contoured to 0.188 SDs. **G,H.** Local resolution map of bUL49.5-TAP(EQ) in the "kinked" conformation. Reconstructions are contoured to 0.135 SDs. **I.** Fourier shell correlation (FSC) curves of the "kinked". **J.** Angular distribution of the final reconstruction. **K.** Map to model fit around the nucleotide binding sites of TAP. Reconstructions are contoured to 0.135 SDs.

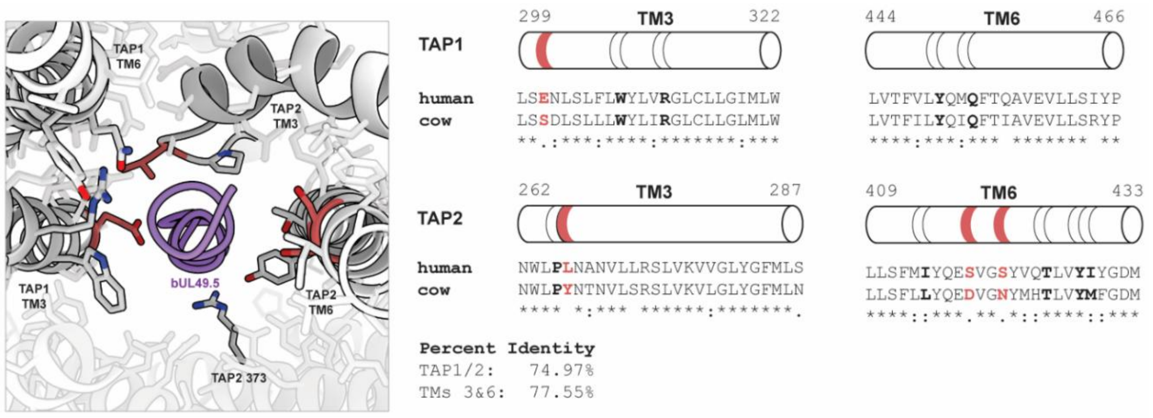

Supplemental Fig. 5. Sequence conservation between human and bovine TAP

(Left) Molecular model showing top view of TAPeq-bUL49.5. A poly-alanine model of bUL49.5 is colored in purple while TAP residues are colored white if identical between human/bovine TAP or otherwise red. Ribbons and select residues on TM3 and TM6 of both TAP1 and TAP2 along with TAP2 R373 are highlighted to depict residues contacting bUL49.5. (Right) Sequence alignments of human and bovine TAP for TM3 and TM6 of both TAP1 and TAP2. Residues in close contact with bUL49.5 are outlined and bolded if identical or similar between species or colored in red if different.

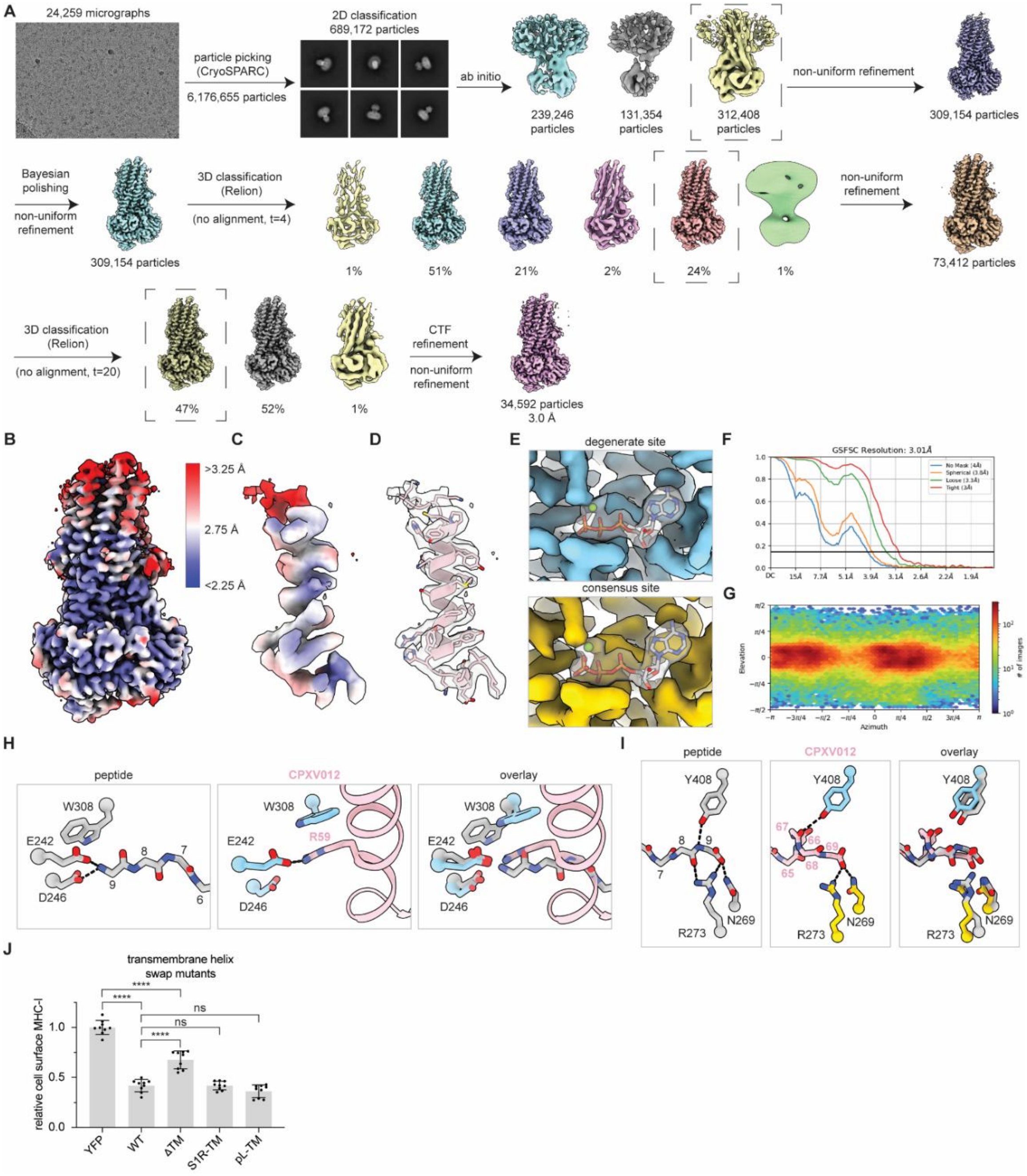

Supplemental Fig. 6. Structural and biochemical analysis of CPXV012-inhibited TAP(EQ).

A. Flowchart of the image processing of CPXV012-inhibited TAP(EQ). **B.** Local resolution map of the final overall complex of CPXV012-inhibited TAP(EQ). **C.** Local resolution map of CPXV012. Reconstructions are contoured to 0.16 SDs. **D.** Map to model fit of CPXV012. Reconstructions are contoured to 0.16 SDs. **E.** Map to model fit around the nucleotide binding sites of TAP. Reconstructions are contoured to 0.3 SDs. **F.** Fourier shell correlation (FSC) curves of the final reconstruction of CPXV012-inhibited TAP(EQ). **G.** Angular distribution of the final reconstruction of CPXV012-inhibited TAP(EQ). **H.** Comparison of peptide (PDB:8T4F) or CPXV012 binding to the TAP N-pocket in the inward-facing state and outward-facing state, respectively. The sidechains of the peptide are hidden for clarity. **I.** Comparison of peptide (PDB:8T4F) or CPXV012 binding to the TAP C-pocket in the inward-facing state and outward-facing state, respectively. The sidechains of

the peptide are hidden for clarity. **J.** The transmembrane helix of CPXV012 is important for localizing the soluble domains to TAP. Flow cytometry analysis of HEK293S cells expressing CPXV012 transmembrane helix variants coupled to YFP with an IRES element. Data represent the relative MHC-I cell surface levels normalized to the YFP only sample and represent three technical replicates of three biological replicates (n=9). Error bars represent the SEM. Statistical significance relative to treatment with wild-type CPXV012 IRES YFP was tested by one-way analysis of variance. ns, not significant; ****P<0.0001.

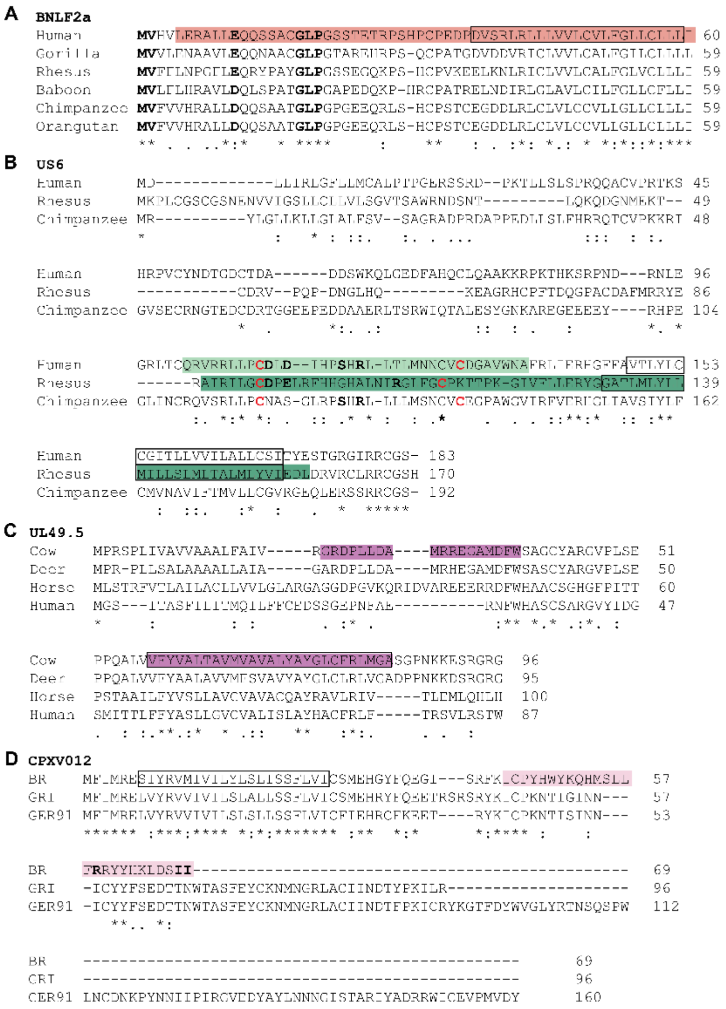

Supplemental Fig. 7. Structural and biochemical analysis of CPXV012-inhibited TAP(EQ).

A. BNLF2a sequence alignment across lymphocryptoviruses. Residues resolved in the cryo-EM density are colored and the transmembrane helix is boxed. Sequences for BNLF2a from human, gorilla, rhesus, baboon, chimpanzee, and orangutan lymphocryptoviruses were retrieved from UniProt using accession numbers P0C739, A2TJU3, Q8UZC8, A2TJU0, A2TJU1, and A2TJU2, respectively. All BNLF2a homologs inhibit TAP. **B.** US6 sequence alignment across cytomegaloviruses. Residues in US6 and rhUS6 resolved in the cryo-EM density are colored light green and sea green, respectively. US6 sequences from human, rhesus, and chimpanzee cytomegaloviruses, were retrieved from UniProt using accession numbers P14334, Q2FAB2, and Q8QRV3, respectively. The cysteines involved in the disulfide bond and the residues shown in Figure 3 are bolded in red and black, respectively. US6 from human and rhesus, but not from chimpanzee, cytomegalovirus inhibit TAP. **V.** UL49.5 sequence alignment across varicelloviruses. Sequences for UL49.5 from cow, deer,s horse, and human were retrieved from UniProt using accession numbers Q77CE4, A0A455JNH1, P28980, and Q0Q872, respectively. UL49.5 from bovine (cow) and cervid (deer) herpesvirus 1 inhibit and degrade TAP. UL49.5 from equine (horse) herpesvirus 1 inhibits but does not degrade TAP. UL49.5 from human herpesvirus 3 does not inhibit or degrade TAP. **D.** CPXV012 sequence alignment across cowpox viruses. Sequences for CPXV012 from cowpox virus strains BR, GRI, and GER91-3 were retrieved from UniProt using accession numbers Q8QN49, G0XT66, and Q0NQ70. CPXV012 from BR, but not from GRI or GER91, inhibits TAP.

	TAP BNLF2a	TAP hUS6	TAP rhUS6	TAP(EQ) bUL49.5 unkinked	TAP(EQ) bUL49.5 kinked	TAP(EQ) CPXV012
	EMD-70314 PDB 9OCG	EMD-70315 PDB 9OCH	EMD-70316 PDB 9OCI	EMD-70241 PDB 9O94	EMD-70246 PDB 9O9D	EMD-70317 PDB 90CJ
**Data collection and processing**						
Magnification	81,000	130,000	130,000	130,000		105,000
Voltage (kV)	300	300	300	300		300
Electron exposure (e–/Å^2)	51	50	50	50		45
Defocus range (μm)	0.8 to 2.5	0.8 to 2.0	0.8 to 1.8	0.8 to 1.8		0.8 to 1.8
Pixel size (Å)	1.08	0.663	0.663	0.743		0.86
Symmetry imposed	C1	C1	C1	C1		C1
Initial particle images (no.)	2,758,634	4,911,717	4,800,952	2,741,436		6,176,655
Final particle images (no.)	35,373	14,445	46,902	168,168	25,389	34,592
Map resolution (Å)	3.7	3.3	3.1	2.5	3.2	3.0
FSC threshold	0.143	0.143	0.143	0.143	0.143	0.143
**Refinement**						
Initial model used (PDB)	8T46	9N63	9N63	9N63	9N63	9N63
Model resolution (Å)	4.1	3.5	3.3	2.6	3.1	3.2
FSC threshold	0.5	0.5	0.5	0.5	0.5	0.5
Map sharpening *B* factor (Å^2)	101.8	48	67.7	54.6	30.8	55.8
Model composition						
Non-hydrogen atoms	7777	8896	9178	8686	8367	9006
Protein residues	1164	1134	1170	1140	1105	1143
Ligands	0	4	4	4	4	4
B factors (Å^2)						
Protein (mean)	145.62	120.42	94.73	83.01	82.81	94.91
Ligands (mean)	N/A	114.02	85.17	61.93	71.09	68.46
R.m.s. deviations						
Bond lengths (Å)	0.011	0.003	0.003	0.003	0.004	0.003
Bond angles (°)	1.648	0.696	0.679	0.614	0.741	0.626
Validation						
MolProbity score	1.20	1.17	1.17	1.03	0.89	1.15
Clashscore	2.20	3.86	3.84	2.43	1.51	3.59
Poor rotamers (%)	0.16	0.00	0.10	0.55	0.47	0.00
Ramachandran plot						
Favored (%)	96.71	98.04	98.45	99.12	99.36	98.33
Allowed (%)	3.29	1.96	1.55	0.88	0.64	1.67
Disallowed (%)	0.00	0.00	0.00	0.00	0.00	0.00

Supplemental Data Table 1. Cryo-EM data collection, refinement, and validation statistics
